# Executive Functioning Training Reduces Cognitive Intra-Individual Variability In Older Adults With HIV

**DOI:** 10.1093/geroni/igaf122.2676

**Published:** 2025-12-31

**Authors:** David Vance, Hathaichanok Phaowiriya, Xueling Zeng, Maryam Rostamvand, Elizabeth Byrd, Raymond Jones, Pamela Bowen, Pariya Fazeli

**Affiliations:** University of Alabama at Birmingham, Birmingham, Alabama, United States; The University of Alabama at Birmingham, Birmingham, Alabama, United States; The University of Alabama at Birmingham, Birmingham, Alabama, United States; The University of Alabama at Birmingham, Birmingham, Alabama, United States; The University of Alabama at Birmingham, Birmingham, Alabama, United States; The University of Alabama at Birmingham, Birmingham, Alabama, United States; The University of Alabama at Birmingham, Birmingham, Alabama, United States; University of Alabama at Birmingham, Birmingham, Alabama, United States

## Abstract

Cognitive training has been shown to enhance cognition in people with HIV (PWH). While these approaches produce some success, none have directly addressed cognitive intra-individual variability (IIV)—fluctuations in cognitive performance within the same test (inconsistency). Higher cognitive IIV is linked to cognitive decline and neurological sequelae in a way standard mean-based cognitive testing does not. The Executive Dyscontrol Hypothesis suggests that impaired executive functioning contributes to greater cognitive IIV. Strengthening executive function may reduce cognitive IIV and improve overall cognitive health. This study examined whether executive functioning training could reduce cognitive IIV in older PWH. In this 2-group baseline/posttest experimental study, we enrolled 118 PWH aged 40 to 70 who were randomized into one of two groups: 1) 20 hours of computerized executive functioning training group, or 2) no-contact control group. At baseline and posttest, participants were assessed on depression, subjective cognitive functioning, and the Connor’s Continuous Performance Test (CCPT-3). The CCPT-3 has two measures of cognitive IIV: 1) HRTSD – hit reaction time standard deviation and 2) variability. In this sample, most were women (52.5%) and African American (85%) and their mean age was 53.87 years; 60 and 58 were in the training group and control group, respectively. Controlling for baseline performance, improvements in HRTSD (F = 3.448, p = 0.067 – trending) and variability (F = 5.163, p = 0.026 – significant) were observed in the treatment group compared to the no-contact control group. Results demonstrate that executive functioning training reduced cognitive IIV; this supports the Executive Dysfunction Hypothesis in a sample at-risk for cognitive problems.

